# Hydrogen-rich water attenuates radiation-induced oral mucositis in mice via antioxidant and gut microbiota-stabilizing effects: a longitudinal study

**DOI:** 10.1080/29933935.2025.2595392

**Published:** 2025-12-10

**Authors:** Zixin Lan, Junyang Chen, Shanwei Lan, Ning Li, Bo Yang, Jin Hou, Xiaojun Yang

**Affiliations:** a Department of Stomatology, Nanfang Hospital, Southern Medical University, Guangzhou, Guangdong Province, China; b Zhujiang Hospital, Southern Medical University, Guangzhou, Guangdong Province, China; c Division of Spine Surgery, Department of Orthopaedics, Nanfang Hospital, Southern Medical University, Guangzhou, China; d Department of Stomatology, Ganzhou People’s Hospital, Ganzhou Hospital–Nanfang Hospital, Southern Medical University, Jiangxi Province, Ganzhou, China

**Keywords:** Radiation-induced oral mucositis, hydrogen-rich water, gut microbiota, short-chain fatty acid

## Abstract

Radiation-induced oral mucositis (RIOM) frequently complicates head and neck radiotherapy, leading to severe pain, compromised nutrition, and often requiring treatment modifications. Although craniofacial-only irradiation is confined to the head and neck region, it can still disrupt gut homeostasis. Mice subjected to head and neck irradiation developed marked epithelial damage in both the oral and intestinal mucosa, as evidenced by pronounced RIOM and diminished barrier integrity. Histological examination revealed substantial mucosal thinning and leukocyte infiltration in the tongue, along with reduced occludin and ZO-1 expression in colonic tissues. Supplementation with hydrogen-rich water (HW) markedly decreased the severity of oral lesions and preserved epithelial thickness, while restoring the expression of tight junction proteins in the colon. Fecal 16S rRNA sequencing showed that radiation alone provoked expansions of *Streptococcus* and *Helicobacter*, coupled with a decline in short-chain fatty acid–producing families (Lachnospiraceae, Ruminococcaceae). In contrast, HW supplementation partially reversed these microbial shifts, which correlated with reduced oral inflammatory markers. Collectively, these findings underscore an oral–gut axis whereby HW fosters mucosal healing through microbiome stabilization and decreased inflammatory stress, suggesting that HW as a promising adjunct for managing head and neck irradiation–related complications.

## Introduction

Radiation-induced oral mucositis (RIOM) is a frequent and debilitating complication of radiotherapy in patients with head and neck cancer. Characterized by painful inflammation of the oral mucosa, RIOM can lead to dysphagia, malnutrition, and treatment interruptions – factors that collectively undermine patients’ quality of life.^1-3^ Its pathogenesis is multifactorial, involving direct cytotoxic damage from ionizing radiation, including apoptosis and excessive production of reactive oxygen species (ROS), as well as indirect inflammatory processes and oral microbiota dysbiosis.^4-8^

Notably, the impact of head and neck irradiation extends beyond the oral cavity, influencing the gut microbiota and promoting potential systemic effects. Clinical data indicate that up to 29% of patients undergoing radiotherapy for head and neck cancer develop moderate to severe diarrhea,^9^ while animal models confirm that gut toxicity can arise even when the intestines are outside the primary radiation field.^10^^,^^11^ Conversely, the gut microbiota is integral to modulating radiotherapy efficacy and toxicity, for instance, germ-free mice exhibit greater resistance to radiation than conventional mice; whereas dysbiosis can exacerbate normal tissue injury.^12-15^ Some studies further associate RIOM with perturbations in the intestinal microbiome,^6-8^^,^^16-18^and probiotics have been explored for enhancing anti-cancer immunity and alleviating treatment-related side effects.^19^ Nonetheless, the precise interplay between gut microbiota shifts and the severity of RIOM remains insufficiently understood.^7^

Existing strategies for mitigating RIOM – such as growth factors, antibiotics, and low-level laser therapy – have demonstrated limited clinical efficacy.^20-27^ Hydrogen-rich water (HW) has emerged as a promising candidate, owing to its well-documented antioxidant properties: it may selectively scavenges cytotoxic ROS, thereby reducing oxidative stress and inflammation.^28-30^ These effects have proven beneficial in various oxidative stress–related conditions, including neurological and cardiovascular diseases.^28-30^ Beyond its antioxidant action, HW also appears to influence gut microbiota composition by promoting beneficial bacterial populations and enhancing overall microbial diversity.^31^^,^^32^ Given that both excess ROS and microbial dysbiosis can aggravate radiation-induced tissue injury, HW’s dual capacity for reducing oxidative stress and stabilizing the microbial ecosystem may be particularly beneficial in RIOM.

In this longitudinal study, we therefore aimed to (i) evaluate HW’s therapeutic efficacy against RIOM, (ii) investigate how gut microbiota structure evolves throughout the course of RIOM, and (iii) determine whether HW’s antioxidant and microbiota-stabilizing actions collectively mitigate radiation-related tissue damage. Ultimately, these insights may guide more targeted strategies to improve clinical outcomes for head and neck cancer patients.

## Materials and methods

### Animals

Male C57BL/6J mice (six weeks old) were housed in a specific pathogen-free (SPF) facility under controlled conditions (23 ± 2 °C, 50 ± 10% relative humidity, and a 12-h light–dark cycle). All procedures were approved by the Experimental Animal Ethics Committee of Nanfang Hospital, Southern Medical University (protocol NFYY-2019-0617), and conducted in strict accordance with institutional guidelines and the ARRIVE (Animal Research: Reporting of In Vivo Experiments) principles to ensure humane treatment and ethical standards throughout the study. The mice were monitored daily for clinical signs and body weight changes. Animals exhibiting >20% body weight loss, severe lethargy, dehydration, or other signs of distress were euthanized immediately to minimize suffering.

In this study, 45 weight-matched mice were randomly assigned to three groups (15 per group): (1) negative control (NC), (2) radiation-only (R), and (3) radiation plus hydrogen-rich water (RHW). The mice were housed 5 per cage to minimize coprophagy-related bias. Dynamic temporal sampling was performed at three distinct time points for morphological and histological examinations, while fecal samples for gut microbiota analysis were collected individually from the same cohort of five mice per group every two days to ensure longitudinal comparability. This design enabled a comprehensive comparison of physiological, histological, and microbial changes over time.

### Hydrogen-rich water treatment

Hydrogen-rich water (HW) was generated three times daily using a proprietary gas–liquid separation system (ZiHe Biology Co., Ltd., China). The dissolved hydrogen concentration was measured immediately before dispensing and was maintained at 1.0–1.4 ppm to ensure a stable hydrogen concentration. The RHW group had ad libitum access to fresh HW starting one day before irradiation (day −1) and continuing throughout the study. In contrast, the NC and R groups received an equivalent volume of standard drinking water at the same time points.

Experimental phases were defined based on gut microbiota diversity, body weight changes, and oral mucositis (OM) scores, with day 13 (D13) separating the aggravation and recovery stages. Further stratification used OM scores: a score of 0–1 prior to D13 was classified as the mild phase, 2–3 as the severe phase, and 0–1 again (but recorded after D13) as the remission phase. This phase structure clarified the role of gut microbiota shifts in RIOM progression and the associated weight fluctuations (Supplementary Figure 1).

### Radiation exposure

After one week of acclimatization, the mice were irradiated on day 0. Anesthesia was induced via intraperitoneal ketamine (100 mg/kg) and xylazine (10 mg/kg) to ensure immobilization and unconsciousness during the procedure. Custom-made lead shields (≥2 mm thick) protected the animals’ bodies, leaving only the oral cavity exposed.

Radiation was delivered using a Faxitron X-ray Multirad irradiator (Faxitron, USA). Each mouse in the R and RHW groups received a single 15 Gy X-ray fraction at a rate of 1.5 Gy/min, as previously reported, targeting the head region (Supplementary Table 1).^11^^,^^33^^,^^34^ The NC group was not irradiated and served as a baseline control.

To prevent ocular damage, erythromycin ointment was applied to the eyes of anesthetized mice prior to shield placement, and immediately after irradiation, the mice were kept on a heated pad for recovery before returning to the vivarium.

### Oral mucositis assessment

Beginning on Day −3, the mice were acclimated to daily oral examinations to reduce interobserver variability. Post-irradiation, the dorsal tongue (tip to the intermolar prominence) was inspected daily by gently exposing the oral cavity. Radiation-induced oral mucositis (RIOM) was scored using the Veterinary Radiation Oncology Group (VRTOG) classification system,^35^ which grades mucositis severity on a scale of 0–3 (Supplementary Table 2), with Grade 3 reflecting the most severe damage. During each assessment, changes such as erythema, focal desquamation, exudation, and ulceration were recorded. Tongue photographs were taken, and two independent, blinded researchers evaluated these images to maintain objectivity in the scoring process.

### Tissue collection and colon length measurement

On days 7, 14, and 21, the mice were humanely euthanized via intraperitoneal injection of pentobarbital (400 mg/kg) following approved protocols. The colon was then isolated from the proximal rectum to the point where it passed under the pelvisternum, ensuring minimal handling and preserving tissue integrity. For length measurement, each colon was placed on a clean, flat surface beside a calibrated ruler; measurements were recorded to the nearest 0.1 cm (from the cecum to the distal rectum) to quantify any radiation-induced gastrointestinal effects.

### Histological analysis

To evaluate the impact of radiation and hydrogen-rich water (HW) on the tongue and intestines, tissues were collected and processed. Tongue tissues were formalin fixed, paraffin embedded (4 μm thick), and stained with hematoxylin and eosin (H&E) to assess epithelial damage, ulceration, and white blood cell infiltration; a modified histological scoring system^36^ was used to quantify epithelial injury (Supplementary Table 3). For intestinal assessments, colons were fixed in 4% paraformaldehyde and stained with both H&E and alcian blue/periodic acid–Schiff (AB/PAS), the latter specifically highlighting goblet cells vital for mucosal barrier function. All the slides were scanned using an SQS-1000 slide scanning imaging system (ShenQiang, China) and analyzed with ImageJ. To minimize interobserver variability, two independent researchers performed all analyses in a blinded manner.

### Immunohistochemistry for tongue tissues

Formalin-fixed, paraffin-embedded tongue sections underwent antigen retrieval in Tris–EDTA buffer (pH 9.0). After being blocked with 10% goat serum, the sections were incubated overnight at 4 °C with primary antibodies against IL-1β (rabbit monoclonal; Abmart, China), TNF-*α* (rabbit monoclonal; Abmart, China), and MPO (rabbit monoclonal; Abcam, UK), each at a ratio of 1:200. The sections were then treated with horseradish peroxidase–conjugated secondary antibodies (Abmart, China), developed with 3,3′-diaminobenzidine tetrahydrochloride hydrate (DAB; Beyotime, China), and counterstained with Mayer’s hematoxylin. After dehydration and mounting, the slides were scanned with an SQS-1000 Slice Scanning Imaging System (ShenQiang, China), and the area of positive staining was quantified via Fiji/ImageJ.

### Immunofluorescence staining for intestinal tight junction proteins

Colon sections underwent antigen retrieval in Tris–EDTA buffer (pH 8.0). Following a 10% goat serum block, the sections were incubated overnight at 4 °C with primary antibodies against occludin and zonula occludens-1 (ZO-1) (rabbit monoclonal; Abcam, UK) at a ratio of 1:200. After washing, the sections were incubated for 1 h at room temperature with FITC-conjugated goat anti-rabbit IgG (1:500; Beyotime, China). Nuclei were counterstained with mounting medium containing DAPI (ab104139, Abcam), followed by dehydration and mounting. Images were acquired on a Nikon Eclipse Ts2-FL inverted microscope (Nikon, Japan), and the percentage area of positive staining was quantified using Fiji/ImageJ.

### Fecal microbiome analysis

Fecal samples were collected from live mice, snap-frozen, and stored at −80 °C. Total DNA was extracted, and the V3–V4 region of the 16S rRNA gene was amplified using barcoded primers (forward: 5′-ACTCCTACGGGAGGCAGCA-3′, reverse: 5′-GGACTACHVGGGTWTCTAAT-3′). PCR was performed at 94 °C for 5  min, followed by 30 cycles of 94 °C for 30  s, 52 °C for 30  s, and 72 °C for 30  s, with a final extension at 72 °C for 10  min. Products were purified (EZNA Gel Extraction Kit, Omega, USA) and sequenced on an Illumina HiSeq2500 according to the manufacturer’s protocols.

Raw reads underwent quality control, denoising, and taxonomic annotation in QIIME 2 to generate an amplicon sequence variants (ASVs) table. Alpha diversity (Shannon index, observed ASVs), beta diversity (Bray–Curtis; unweighted/weighted UniFrac), and principal coordinate analysis (PCoA) were calculated as described previously.^37^^,^^38^ Differential features among groups were identified via linear discriminant analysis effect size (LEfSe, LDA threshold = 2.0).^39^^,^^40^ “First-distances” analysis (QIIME2-longitudinal) was conducted to evaluate beta diversity differences between successive samples from the same subject. Spearman’s rank correlations were performed in R (v4.4.0), and co-abundance network analysis relied on the same software, with filtering at 0.0005 (filter_thres) and a correlation cutoff of 0.4 (COR_cut). Statistical analysis of genus-level relative abundances was also carried out in R (v4.4.0).

### Statistical analysis

All the statistical analyses were carried out in GraphPad Prism 10.1.2. Parametric data were evaluated using Student’s t-test, one-way or two-way analysis of variance (ANOVA), or mixed-effects models, whereas nonparametric data were analyzed via Mann–Whitney U or Kruskal–Wallis tests. The results are presented as the mean ± standard error of the mean (SEM), and *p* < 0.05 was deemed significant. Figures display significance as *p < 0.05, **p < 0.01, ***p < 0.001, and ****p < 0.0001.

## Results

### Hydrogen-rich water alleviates radiation-induced oral mucositis and intestinal damage

**Figure 1.**
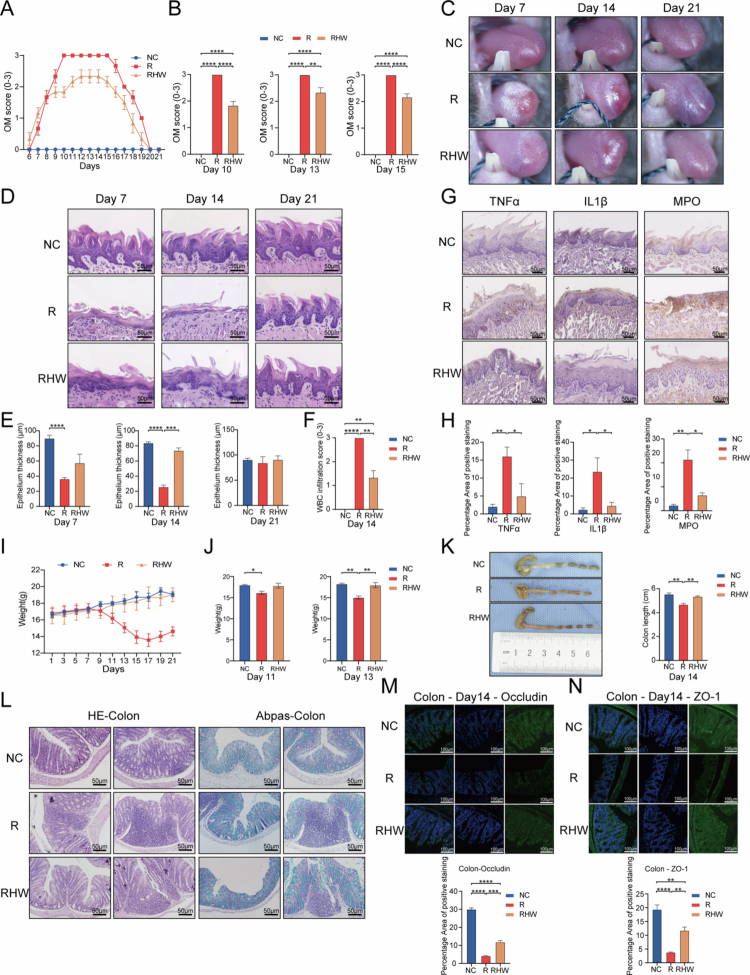
Hydrogen-rich water attenuates radiation-induced oral mucositis and intestinal damage in mice. (A) Oral mucositis (OM) severity scores from day 6 to day 21 post-irradiation for the negative control (NC), radiation-only (R), and radiation + hydrogen-rich water (RHW) groups. (B) OM severity scores on days 10, 13, and 15 (Bonferroni test). (C) Representative tongue images from the NC, R, and RHW groups on days 7, 14, and 21, showing ulcer-like lesions and tissue damage. (D) Hematoxylin and eosin (H&E) staining of tongue sections on days 7, 14, and 21, indicating epithelial thinning and white blood cell (WBC) infiltration. (E) Epithelial thickness of the tongue tip in the NC, R, and RHW groups (mixed-effects analysis/Bonferroni test). (F) WBC infiltration scores at the tongue tip (Kruskal–Wallis test). (G) Immunohistochemical staining for TNF-*α*, IL-1β, and MPO in the tongue tip. (H) Quantification of TNF-*α*, IL-1β, and MPO levels in the tongue tip (Kruskal–Wallis test). (I) Body weight changes postirradiation. (J) Body weight on day 11 for the NC, R, and RHW groups (mixed-effects analysis/Bonferroni test). (K) Colon length on day 14 (mixed-effects analysis/Bonferroni test). (L) H&E and Alcian blue–periodic acid–Schiff (AB-PAS) staining of the colon on day 14. (M) Immunofluorescence staining for occludin, showing the percentage of positive-stained area. (N) Immunofluorescence staining for zonula occludens-1 (ZO-1), indicating the percentage of positive-stained area. *N* = 4–5 per group, representing the number of mice sampled at each time point. The data were averaged for each set of 4–5 mice to assess temporal variations within each group. Scale bars = 100 μm. Abbreviations: NC, negative control; R, radiation; RHW, radiation + hydrogen-rich water; OM, oral mucositis; TOT, tip of the tongue; ZO-1, zonula occludens-1. Statistical significance is marked as *p < 0.05, **p < 0.01, ***p < 0.001, and ****p < 0.0001.

Mice subjected to single-dose head and neck irradiation developed pronounced RIOM that peaked in severity on day 10. Throughout the observation period, the mice that received hydrogen-rich water (RHW group) presented consistently lower oral mucositis severity scores compared with irradiated-only counterparts (R), especially evident from day 9 onwards through day 15 (Figure 1A,B). Gross examination confirmed these findings, showing clearly reduced mucosal lesions in RHW mice (Figure 1C). Histological analyses further demonstrated notable epithelial thinning and intense white blood cell (WBC) infiltration in the R group at days 7 and 14, whereas these changes were only mild in the RHW group (Figure 1D−F).

Consistently, irradiation markedly elevated TNF-*α*, IL-1β, and MPO in the dorsal tongue mucosa, increasing inflammation and oxidative stress. Although we did not directly quantify ROS, the levels of these mediators were substantially lower in RHW mice than in control mice on day 14 (Figure 1G,H), aligning with the proposed antioxidant-like effects of hydrogen. This reduction in pro-inflammatory markers likely contributed to less tissue damage and faster mucosal recovery.

Despite abdominal shielding, the R group displayed severe diarrhea and significant weight loss starting around day 7 and persisting through day 17, indicating broader systemic effects. In contrast, the RHW group maintained body weights comparable to the NC group, with no pronounced decline observed, a trend that remained evident on days 11 and 13 (Figure 1I,J).

Parallel to the reduction in weight loss, colon length and histological evaluations revealed milder intestinal damage in RHW mice. Specifically, these animals exhibited better retention of the colonic mucosal architecture, reduced inflammatory cell infiltration, and relatively stable goblet cell counts compared to the R group (Figure 1K,L). Moreover, the levels of the intestinal tight junction proteins occludin and ZO-1 were markedly reduced in the R group but remained relatively high in the RHW mice group (Figure 1M,N). This preservation of barrier integrity suggests that HW not only dampens local inflammatory responses but also fosters a more favorable gut environment – possibly by limiting oxidative damage and stabilizing the microbial milieu. Given these morphological and barrier-protective benefits, we next investigated whether HW-driven improvements might be tied to shifts in the gut microbial community.

### Hydrogen-rich water preserves gut microbiota diversity during RIOM progression and recovery

**Figure 2.**
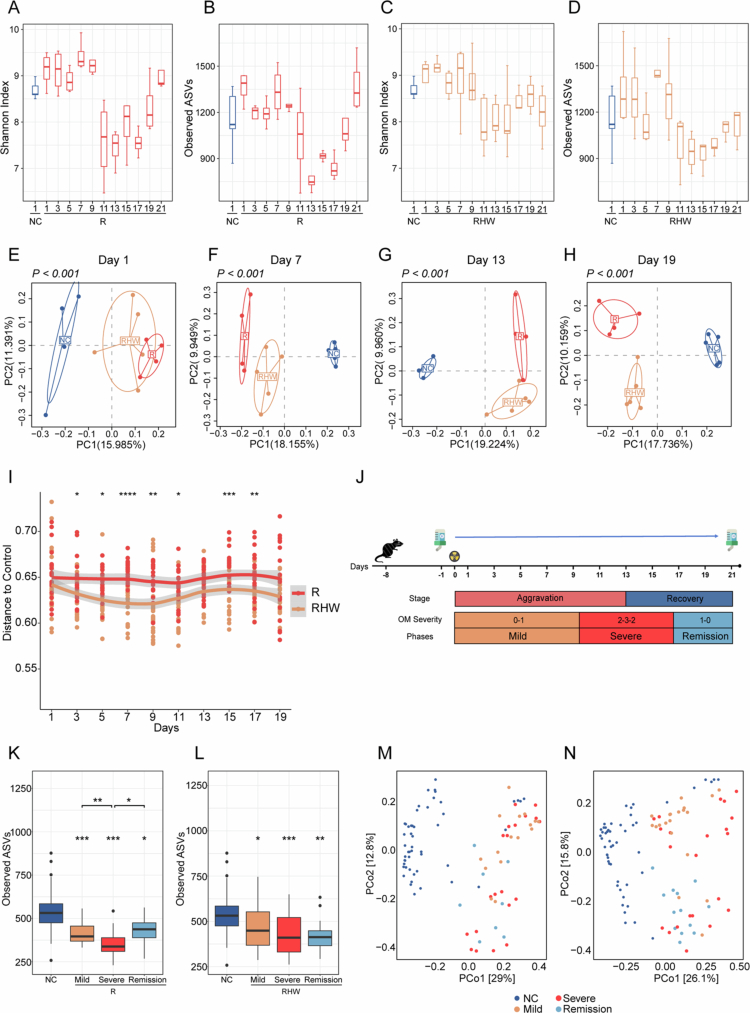
Hydrogen-rich water preserves gut microbiota diversity during RIOM progression and recovery. (A, B) Comparison of alpha diversity indices, including the Shannon index (A) and observed ASVs (B), between NC and R. (C, D) Comparison of alpha diversity indices, including the Shannon index (C) and observed ASVs (D), between NC and RHW. (E–H) Principal coordinate analysis (PCoA) based on unweighted UniFrac distances at days 1, 7, 13, and 19, respectively; each point represents a single sample color-coded by group, with axis percentages indicating the variance explained. (I) Scatterplot illustrating changes over time in the unweighted UniFrac distance from the NC for R and RHW mice; each subject’s longitudinal data are connected, and statistical significance (R vs. RHW) is shown for each day. (J) Schematic of the experimental design. (K, L) Alpha diversity (Shannon index) among NC and the mild, severe, and remission phases for R (K) and RHW (L). (M, N) PCoA plots based on Bray–Curtis distance comparing R (M) or RHW (N) with NC across mild, severe, and remission phases; each circle denotes a single sample color-coded by group, and axis percentages indicate the variance explained. Statistical significance was assessed using the Wilcoxon rank-sum test unless otherwise noted. For (K, L), significance vs. NC is indicated above each RIOM phase. The significance levels are *p < 0.05, **p < 0.01, ***p < 0.001, and ****p < 0.0001.

To explore how head and neck irradiation affects gut bacterial communities – and whether HW can mitigate such changes – we conducted 16S rRNA gene sequencing of fecal samples. Alpha diversity analysis revealed pronounced fluctuations in the R group: a steep decline around day 11, the lowest values on day 13, and a partial rebound by day 21 (Figure 2A,B). The RHW group followed a broadly similar downward trend but with less drastic reductions in alpha diversity (Figure 2C,D). Beta diversity assessments also revealed significant differences among all groups (Figure 2E−H, Supplementary Figure 2A−F). Both the R and RHW groups initially deviated from the NC group but gradually moved closer over time. Notably, at nearly every time point, the RHW group maintained a smaller distance from the NC than the R group, reflecting a closer approximation to the control condition (Figure 2I).

These observations pinpoint day 13 as a critical transition from RIOM aggravation to recovery, coinciding with the lowest observed alpha diversity in both groups. To explore this pattern more thoroughly, we categorized individual mouse samples into “mild,” “severe,” or “remission” phases based on each animal’s day-specific OM severity scores (Figure 2J). During the severe phase, both irradiated groups presented significant alpha diversity reductions, but the R group’s decline was more pronounced – suggesting that HW may help protect against bacterial community collapse (Figure 2K,L). Although alpha diversity modestly rebounded in both groups during the remission phase, neither fully returned to NC levels.

Beta diversity analysis revealed distinct clustering patterns between the R and RHW groups compared to NC group across the progression of RIOM (Figure 2M–N). In the R group (Figure 2M), samples during peak mucositis formed a cluster substantially separated from the NC group, reflecting a pronounced shift in community composition. Even as the mice progressed toward remission, the points only partially moved back toward the NC cluster, indicating incomplete restoration of the gut microbial structure. In contrast, the RHW group (Figure 2N) consistently remained closer to the NC group, especially during the mild and remission phases. Though these samples also diverged from NC at peak mucositis, they converged more strongly toward the control profile over time, suggesting that HW treatment helped preserve a baseline-like community.

These data highlight a strong link between oral mucositis severity and gut microbiota disruption, mirroring systemic oxidative and inflammatory stress induced by radiation. By stabilizing alpha diversity and promoting a microbiota closer to the healthy baseline, HW administration may counteract radiation-induced dysbiosis, supporting its proposed roles in antioxidant and anti-inflammatory effects.

### Hydrogen-rich water influences key microbial taxa across RIOM stages

**Figure 3.**
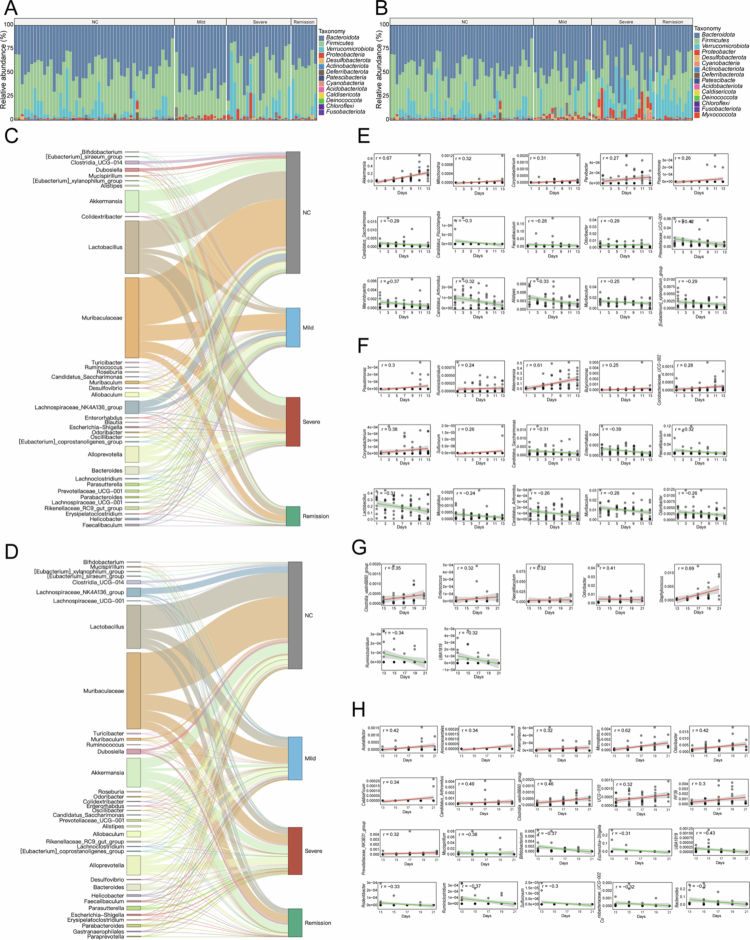
Hydrogen-rich water alters key microbial taxa across RIOM stages. (A, B) Relative abundances of gut microbes at the phylum level among NC mice and those in the mild, severe, and remission phases at R (A) and RHW (B). (C) Dynamic changes in genus-level abundance across NC and the three phases in the R group. (D) Dynamic changes in genus-level abundance across NC and the three phases in the RHW group. (E) Scatter plots with trend lines showing significant positive or negative correlations for 15 bacterial genera with time postradiation in the R group’s aggravation stage. (F) Scatter plots with trend lines showing similar correlations for 15 bacterial genera in the aggravated stage of the RHW group. (G) Scatter plots with trend lines depicting correlations for 7 bacterial genera in the R group’s recovery stage. (H) Scatter plots with trend lines depicting correlations for 20 bacterial genera in the RHW group’s recovery stage.

At the phylum level, Firmicutes and Bacteroidetes remained dominant across all treatment groups. In the R group, Proteobacteria increased during the severe phase of RIOM, while Verrucomicrobiota followed a biphasic pattern – decreasing in the mild phase before rising again at peak disease (Figure 3A). In contrast, the mice that received hydrogen-rich water presented significant enrichment of Verrucomicrobiota during remission, a shift not observed in the R group (Figure 3B).

At the genus level, *Lactobacillus* and *Akkermansia* were both prominent in the healthy NC controls. In the R group, however, each peaked during the severe phase but fell to comparatively lower levels in the mild and remission phases (Figure 3C). In contrast, the RHW group maintained more stable *Lactobacillus* and *Akkermansia*, with *Akkermansia* remaining higher in remission than in the R group (Figure 3D). Although *Lactobacillus* and *Akkermansia* expanded briefly in the R group – likely in a short-lived effort to counteract dysbiosis – their more consistent, higher-level presence in RHW-treated mice suggests a sustained protective role. By preserving these beneficial genera throughout RIOM progression, HW may support both gut barrier function and anti-inflammatory pathways, ultimately promoting a more stable, health-aligned microbiota.

Spearman’s rank correlation analyses revealed distinct temporal patterns in the aggravation (pre-day 13) and recovery (post-day 13) stages (Figure 3E–H). In the R group, *Akkermansia* and *Pseudomonas* were positively correlated with time during aggravation, while several SCFA-producing genera, such as Lachnospiraceae and Ruminococcaceae, were negatively correlated. (Figure 3E). Although the RHW group presented some overlapping trends, SCFA producers such as *Butyricimonas* and *Ruminiclostridium* were positively correlated (Figure 3F). In the recovery phase, the R group presented positive correlations for *Staphylococcus* and *Enterococcus* – both commonly considered opportunistic in the gut – whereas *Ruminiclostridium* presented a negative correlation (Figure 3G). In contrast, RHW mice presented positive correlations with certain Lachnospiraceae, Ruminococcaceae, and Clostridiaceae members, along with a negative correlation with the potentially pathogenic *Escherichia–Shigella* (Figure 3H).

Collectively, these findings underscore HW’s selective modulation of key bacterial populations across RIOM aggravation and recovery. In particular, shifts in *Akkermansia* and SCFA-producing families (e.g., Lachnospiraceae, Ruminococcaceae) align with a more balanced gut environment – an outcome that may help alleviate oxidative or inflammatory stress under radiation-induced injury.

### Hydrogen-rich water reshapes microbial networks and composition following RIOM

To investigate how head and neck irradiation influences gut microbial communities – particularly short-chain fatty acid (SCFA) producers – and whether hydrogen-rich water (HW) can mitigate these disruptions, we conducted a detailed co-abundance network analysis. This method identifies ecological clusters of bacterial genera based on their correlated relative abundances.

**Figure 4.**
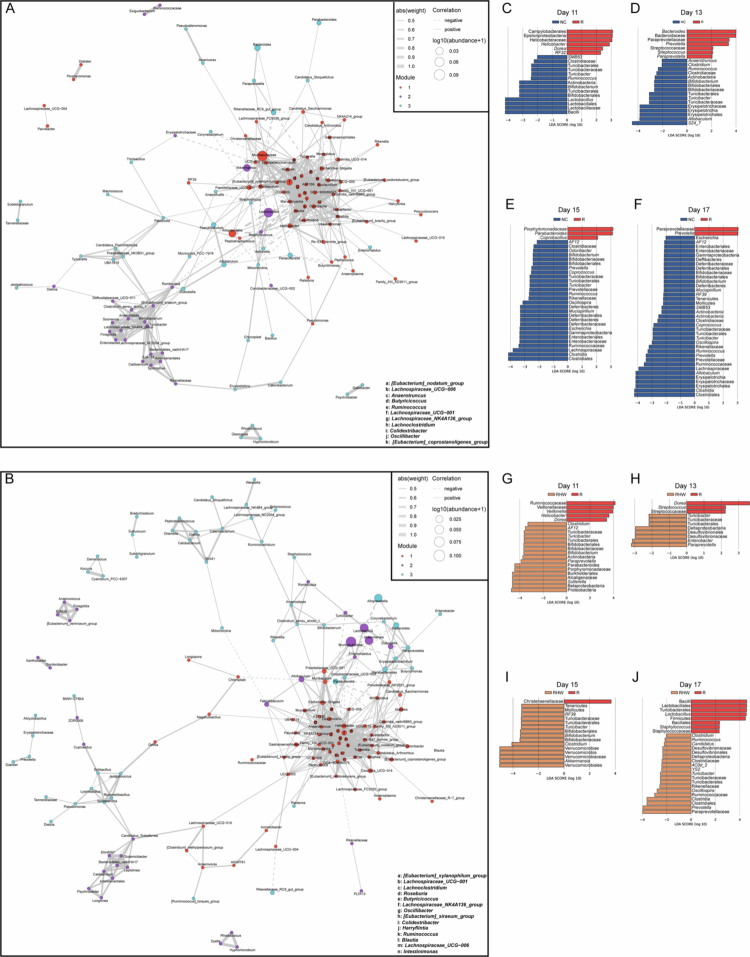
Hydrogen-rich water reshaped microbial networks and compositions following RIOM. (A, B) Co-abundance networks of significantly correlated gut bacterial genera across the entire mucositis progression in the R (A) and RHW (B) groups. Each node represents a bacterium, scaled by mean relative abundance; remove isolated nodes (those not connected to any other nodes); solid lines denote positive correlations, dotted lines denote negative correlations, and line thickness reflects correlation strength. (C–F) LEfSe analysis comparing the R and NC groups during aggravation (day 11 for C, day 13 for D) and recovery (day 15 for E, day 17 for F). A positive LDA score indicates R-enriched taxa, whereas a negative score indicates NC-enriched taxa (only taxa with LDA > 2 are shown). (G–J) LEfSe analysis comparing the R and RHW groups during aggravation (day 11 for G, day 13 for H) and recovery (day 15 for I, day 17 for J). A positive LDA score indicates R-enriched taxa, while a negative score indicates RHW-enriched taxa (only taxa with LDA > 2 are shown). LEfSe, linear discriminant analysis effect size; LDA, linear discriminant analysis.

In the irradiation-only (R) group (Figure 4A, Supplementary Figure 3), Cluster 1 included 65 bacterial taxa, among which 33 were known or putative SCFA producers, primarily within the Lachnospiraceae, Ruminococcaceae, and Clostridiaceae families.^37-40^ In contrast, the RHW group’s Cluster 1 was larger, comprising 68 taxa (Figure 4B, Supplementary Figure 4), including 36 SCFA-associated genera. Notably, the RHW group displayed greater representation and tighter clustering of these beneficial genera, especially within Lachnospiraceae and Ruminococcaceae, suggesting an enhanced ecological network and potentially elevated SCFA production capacity compared to the R group.

Furthermore, Cluster 1 in the RHW group demonstrated a strong positive correlation with Cluster 2, which was enriched in beneficial genera such as *Allobaculum* and *Faecalibacterium* – both recognized for their robust anti-inflammatory properties.^41^ The increased size and connectivity of these clusters in the RHW group suggest that HW intervention effectively reinforces beneficial microbial interactions, enhancing gut microbial resilience and barrier integrity in the context of irradiation-induced dysbiosis.

Because co-abundance data alone did not provide a complete distance between the SCFA-producing taxa in the R and RHW groups, particularly during the severe and remission phases, we employed LEfSe analysis to identify specific changes in bacterial genera. To differentiate solely the microbial shifts resulting from radiation exposure, we first compared the R group to healthy NC mice. During the aggravation phase, the R samples showed significant divergence from NC, marked by an enrichment of *Helicobacter*, *Bacteroides*, *Streptococcus*, *Coprobacillus*, and *Paraprevotella*. In contrast, the NC group consistently presented increased levels of beneficial genera such as *Lactobacillus*, *Bifidobacterium*, and *Ruminococcus*. In the recovery phase, the R group became predominantly populated by *Parabacteroides* and *Coprobacillus*, whereas the NC group maintained a more diverse microbial community enriched in Clostridiaceae, Lachnospiraceae, Bifidobacteriaceae, Ruminococcaceae, Erysipelotrichaceae, and Rikenellaceae (Figure 4C–F, Supplementary Figure 5A, B) – each of which has been shown to contribute to SCFA production in mice.^42-47^

We next examined whether HW consumption could counteract these dysbiotic features by comparing the R and RHW groups directly. In the aggravation phase, R resulted in sequential increases in *Helicobacter* and *Streptococcus*, whereas RHW resulted in increases in *Bifidobacterium* and *Turicibacter* (Figure 4G,H, Supplementary Figure 5C). During recovery, R was enriched in *Staphylococcus*, but RHW was enriched in *Akkermansia*, *Bifidobacterium*, and multiple SCFA-producing families (Clostridiaceae, Ruminococcaceae, Rikenellaceae and Paraprevotellaceae). Notably, the persistent presence of certain potentially pathogenic genera – such as *Streptococcus*, *Helicobacter*, or *Staphylococcus* – in R underscores ongoing dysbiosis, while these taxa are less abundant or suppressed in RHW, suggesting a protective shift that may alleviate post-radiation stress. Consequently, the RHW samples more closely resembled the NC samples, indicating that HW fosters a gut microbiota closer to the baseline state.

To further investigate the potential functional roles of the gut microbiota, we performed Phylogenetic Investigation of Communities by Reconstruction of Unobserved States 2 (PICRUSt2) analysis to predict microbial metabolic capacities. As shown in Supplementary Figure 6, several SCFA-related pathways – including pyruvate metabolism, propanoate metabolism, and fatty acid biosynthesis – were significantly enriched in the RHW group compared to the R group, suggesting a shift toward increased SCFA production potential following hydrogen-rich water intervention.

Collectively, these findings demonstrate that HW significantly reshaped the gut microbial network in irradiated mice by enhancing SCFA-producing taxa and mitigating dysbiosis. Although additional mechanistic studies (e.g., direct SCFA or ROS quantification) are necessary to confirm causality, our results reinforce that stabilizing the gut microbiota is integral to the radioprotective benefits of HWs.

## Discussion

This study demonstrated that hydrogen-rich water (HW) exerts notable protective effects against radiation-induced oral mucositis (RIOM) in mice while also mitigating intestinal injury and stabilizing the gut microbiota. In what follows, we integrate our results with existing literature to reveal the complex mechanisms at play – focusing on oxidative stress, inflammation, and microbial alterations – and consider how these findings might inform clinical strategies in radiotherapy.

First, our data confirm that head and neck irradiation triggers significant oxidative stress in the oral mucosa, as reflected by elevated inflammatory markers and pronounced histological damage in both irradiated groups.^48^^,^^49^ However, the marked reduction in RIOM severity under HW treatment suggests a dual antioxidant and anti-inflammatory mechanism. Hydrogen molecules in HW may selectively scavenge cytotoxic reactive oxygen species (ROS), thereby minimizing ROS-mediated cellular damage and preventing overactivation of inflammatory pathways (e.g., NF-κB).^50^^,^^51^ Additionally, lowering ROS indirectly decreases pro-inflammatory cytokines such as TNF-*α* and IL-1β, as shown by our immunohistochemical analyses. Similar radioprotective outcomes have been observed in other oxidative stress–related disease models, implying that the benefits seen in RIOM may stem from the broader redox-regulating properties of HWs.^52^^,^^53^

Second, our findings show that localized head and neck irradiation can produce systemic repercussions, notably in the gut microbiota.^54^ Despite shielding most of the body, radiation still triggered intestinal injury and dysbiosis, supporting the concept of a gut–oral axis.^55-57^ The irradiated-only group experienced a sharp decline in alpha diversity – especially during severe phases – coupled with significant deviations in beta diversity, mirroring a destabilized microbial community that coincided with peak mucosal damage. Indeed, severe oral mucositis can impact food intake due to pain and dysphagia, which could potentially influence weight and microbial changes.^33^^,^^36^^,^^58^ In contrast, the RHW group preserved higher alpha diversity, which aligns with prior reports linking greater microbial diversity to stronger mucosal defenses and enhanced resilience against inflammatory insults.^59-61^

Third, the preservation of beneficial genera in the RHW group emerges as integral to mucosal protection. Notably, *Lactobacillus* remained relatively stable and abundant – consistent with its known roles in immunomodulation and barrier support.^62^^,^^63^ *Akkermansia*, another key taxon stabilized by HW, is associated with improved barrier function via mucin degradation and synergy with other microbes.^64^^,^^65^ The collective presence of these bacteria likely aids epithelial repair and helps regulate immune responses, thus limiting mucosal injury. Furthermore, *Bifidobacterium* was enriched in HW-treated mice, presumably contributing to short-chain fatty acid (SCFA) production, particularly acetate, which may support local immune regulation.^66-68^ Such increases in beneficial bacteria paralleled the increase in classic SCFA-producing families (e.g., Lachnospiraceae, Ruminococcaceae, Clostridiaceae), reinforcing epithelial integrity and dampening inflammatory pathways.^69-71^ SCFAs – especially butyrate – support epithelial cohesion (occludin, ZO-1) and modulate pro-inflammatory signaling,^72^ hinting a synergy between the antioxidant effects of HWs and the SCFA-rich microbiome in alleviating both oral and intestinal radiation injury.

Fourth, dysbiosis in the radiation-only group coincided with the proliferation of potentially pathogenic genera (*Helicobacter*, *Streptococcus*, *Staphylococcus*) that can drive further inflammation or predisposition to secondary infections. In contrast, HW-treated animals suppressed these harmful taxa, indicating that HW not only foster beneficial microbes but also restricts opportunistic species. This shift toward a more balanced ecosystem – marked by reduced inflammatory cytokines and fewer pathogenic bacteria – appears to underlie the improved outcomes in HW mice.

Taken together, these findings highlight the twofold advantage of hydrogen-rich water in the setting of head and neck radiotherapy: (1) scavenging ROS and moderating inflammation, and (2) preserving a healthier gut microbiome enriched in SCFA-producing, barrier-supportive genera. This synergy can interrupt the self-perpetuating inflammatory cycle that frequently worsens mucosal damage under radiation stress. Moreover, we noted a striking decline in potentially pathogenic taxa in HW-treated mice, suggesting that a stabilized microbiome can provide further protection against secondary infections or dysbiosis-driven exacerbations of RIOM.

The broader clinical relevance of these observations becomes evident in light of the limited efficacy and potential drawbacks of existing RIOM treatments, including growth factors, antibiotics, and low-level laser therapy.^73^^,^^74^ Our data indicate that HW addresses multiple aspects of radiation-induced injury – both oxidative and microbial – and may thus serve as safe and effective adjuncts in head and neck cancer radiotherapy. Notably, a central strength of our study lies in its longitudinal design, allowing us to track both RIOM severity and microbiota dynamics over time. With 16S rRNA gene sequencing, we identified essential SCFA-producing families tied to mucosal defense, spotlighting a crucial oral–gut axis in radiotherapy outcomes.

Nevertheless, additional investigations are needed to fully elucidate these mechanistic links. Future directions include direct SCFA and ROS quantification, fractionated irradiation models, immune profiling, and multi-omics approaches (e.g., metabolomics, metagenomics) to determine precisely how HW’s antioxidant properties and microbiota-stabilizing effects converge to protect oral and intestinal tissues. Importantly, fractionated irradiation protocols – which better reflect clinical radiotherapy schedules – should be adopted to assess whether HW’s protective effects persist under more translationally relevant conditions. Given that only male mice were used in this study to reduce biological variability, future work should incorporate both sexes to increase generalizability. Additionally, causal approaches such as fecal microbiota transplantation or germ-free mouse models are warranted to establish the role of the microbiota directly in modulating RIOM progression. Despite these questions, our results emphasize the importance of combining antioxidant support with microbiome stabilization – particularly by fostering beneficial SCFA producers – to enhance patient outcomes in head and neck cancer radiotherapy.

In conclusion, this study shows that hydrogen-rich water confers notable radioprotection in a murine model of irradiation-induced oral mucositis by reducing inflammatory stress and cultivating a healthier gut microbial profile. Although further mechanistic work is warranted to establish direct causal pathways, these findings – along with evidence that HWs do not promote tumor growth^75-77^ – reinforce the potential of HWs as a multi-targeted strategy for mitigating radiation-induced complications in oncology. By highlighting the interplay between the gut microbiome and oral health, our work adds to the emerging consensus that harnessing the gut microbiome offers a promising approach for alleviating RIOM and improving the effectiveness of radiotherapy.

## Supplementary Material

Supplementary material Supplementary material

## Data Availability

The raw sequence data for all samples used in this study were deposited in the NCBI repository BioProject PRJNA1365002 (https://www.ncbi.nlm.nih.gov/bioproject/PRJNA1365002).
