# Supplementary material for: Hydrogen-rich water attenuates radiation-induced oral mucositis in mice via antioxidant and gut microbiota-stabilizing effects: a longitudinal study
Source: Gut Microbes Rep. 2025 Dec 10;2(1):2595392. doi: 10.1080/29933935.2025.2595392 (PMC12940104; doi:10.1080/29933935.2025.2595392)
Supplement: Supplementary material [file KGMR_A_2595392_SM9679.docx]

**Hydrogen-Rich Water Attenuates Radiation-Induced Oral Mucositis in Mice via Antioxidant and Gut Microbiota-Stabilizing Effects: A Longitudinal Study**

Zixin Lan^1,2†^, Junyang Chen^1†^, Shanwei Lan^3^, Ning Li^1^, Bo Yang^1^*, Jin Hou^1^*, and Xiaojun Yang^1,4^*

^1^ Department of Stomatology, Nanfang Hospital, Southern Medical University, 510515 Guangzhou, Guangdong Province, China

^2^ Zhujiang Hospital, Southern Medical University, 510280 Guangzhou, Guangdong Province, China

^3^ Division of Spine Surgery, Department of Orthopaedics, Nanfang Hospital, Southern Medical University, 510515 Guangzhou, China.

^4^ Department of Stomatology, Ganzhou People’s Hospital, Ganzhou Hospital-Nanfang Hospital, Southern Medical University, Jiangxi Province, 341000 Ganzhou, China.

^†^ These authors share the first authorship.

***Corresponding authors**:

Prof. Xiaojun Yang

Address: No.1838, North Guangzhou Avenue, Guangzhou, China.

E-mail: [yangxiaojun@smu.edu.cn](mailto:yangxiaojun@smu.edu.cn)

Prof. Jin Hou

Address: No.1838, North Guangzhou Avenue, Guangzhou, China.

E-mail: [houjin@smu.edu.cn](mailto:houjin@smu.edu.cn)

Dr. Bo Yang

Address: No.1838, North Guangzhou Avenue, Guangzhou, China.

E-mail: [ybb7@163.com](mailto:ybb7@163.com)

**This file includes:**

Appendix A: Methods and materials

Appendix B: Tables and figures

**Appendix A**

**Methods and materials**

***Hydrogen-Rich Water Treatment***

The RHW group was provided with ad libitum access to fresh hydrogen-rich water (HW), beginning one day before radiation exposure (day -1) and continuing throughout the experimental period. To control for potential confounding effects of water intake, both the NC and R groups were given an equivalent volume of standard drinking water at the same time points as the RHW group. The study divided the experimental phases based on gut microbiota diversity, body weight fluctuations, and oral mucositis (OM) scores. The phases were categorized into an aggravation stage and a recovery stage, with day 13 (D13) as the dividing point. OM scores were used to further define the phases: scores of 0-1 before D13 were categorized as the mild phase, scores of 2-3 as the severe phase, and scores of 1-0 after D13 as the remission phase. This stratification aimed to clarify the role of the gut microbiota in the progression of radiation-induced oral mucositis and its relationship with changes in body weight (Supplementary Figure 1).

***Tissue Collection and Colon Length Measurement***

At the conclusion of the treatment schedule on days 7, 14, and 21, mice were humanely euthanized by intraperitoneal injection of pentobarbital (400 mg/kg) according to established protocols. The colon was carefully isolated, extending from the proximal rectum to its passage under the pelvisternum, ensuring the integrity of the organ throughout the dissection process.

For colon length measurement, a standardized procedure was employed. The isolated colon was placed on a clean, flat surface alongside a calibrated ruler. Measurements were taken from the proximal end of the cecum to the distal end of the rectum, with readings recorded to the nearest 0.1 cm. This method provided a precise and quantitative assessment of colon length for each mouse, serving as an indicator of potential radiation-induced gastrointestinal alterations.

***Immunohistochemistry for Tongue Tissues***

Macroscopic and histological analyses were performed to assess the effects of radiation and hydrogen-rich water (HW) treatment on tongue and intestinal tissues. Tongue tissues were collected to evaluate injury and inflammatory markers, while intestinal tissues were examined to assess radiation-induced effects on intestinal barrier integrity.

For tongue tissue analysis, samples were processed into 4 μm thick formalin-fixed paraffin-embedded sections and stained with hematoxylin and eosin (H&E) to evaluate epithelial thickness, mucosal ulceration, and white blood cell (WBC) infiltration. A modified histological scoring system [1] was used to quantify epithelial damage (Supplementary Table 3).

For the intestinal analysis, the colon was measured, fixed in 4% paraformaldehyde, and processed for both H&E and Alcian Blue/Periodic Acid Schiff's (AB/PAS) staining. The AB/PAS staining specifically targeted intestinal goblet cells (GCs), a critical component of the intestinal mucosal barrier. Colon sections were stained with AB/PAS and counterstained with hematoxylin for visualization.

All histological slides were scanned using the Slide Scanning Imaging System SQS-1000 (ShenQiang, China) and analyzed with ImageJ software. To ensure objectivity and minimize interobserver variability, all analyses were conducted by two independent researchers in a blinded manner.

***Immunofluorescence Staining for Intestinal Tight Junction Proteins***

Paraffin sections were subjected to antigen retrieval using Tris-EDTA buffer (pH 8.0). After blocking with 10% goat serum, the sections were incubated overnight at 4°C with primary antibodies specific for occludin and zonula occludens-1 (ZO-1) (Abcam, Cambridge, UK), both diluted at 1:200 in blocking buffer. Following this, sections were incubated with FITC-conjugated goat anti-rabbit IgG (Beyotime, China) at a dilution of 1:500 for 1 hour at room temperature. Nuclei were counterstained with a mounting medium containing 4',6-diamidino-2-phenylindole (ab104139, Abcam).

Afterward, sections were dehydrated, cleared, and mounted. Images were captured using a conventional inverted microscope (Nikon, Eclipse Ts2-FL, Japan). The percentage area of positive staining for each tight junction marker was quantified using Fiji/ImageJ software, providing a quantitative assessment of the expression levels of these proteins.

***Fecal Microbiome Analysis***

Fecal samples were collected from live mice, snap-frozen, and stored at −80°C. DNA extraction was performed, and the V3-V4 region of the 16S rRNA gene was amplified by polymerase chain reaction (PCR) using barcoded primers: Forward (5'-ACTCCTACGGGAGGCAGCA-3') and Reverse (5'-GGACTACHVGGGTWTCTAAT-3'). PCR conditions included an initial denaturation at 94°C for 5 minutes, followed by 30 cycles of 94°C for 30 seconds, 52°C for 30 seconds, and 72°C for 30 seconds, with a final extension at 72°C for 10 minutes. PCR products were purified using an EZNA Gel Extraction Kit (Omega, USA) and sequenced on the Illumina HiSeq2500 platform according to the manufacturer’s instructions.

The 16S rRNA sequencing data underwent quality control, denoising, and taxonomic annotation, followed by construction of an amplicon sequence variants (ASVs) table using Qiime2. Alpha diversity was assessed using the Shannon index and Observed ASVs, while beta diversity was evaluated using Bray-Curtis, unweighted, and weighted UniFrac distance metrics. Principal coordinate analysis (PCoA) based on UniFrac metrics was also performed.

To identify differential features between groups, linear discriminant analysis effect size (LEfSe) was conducted [2], with the threshold for discriminative features set at a logarithmic Linear Discriminant Analysis (LDA) score of 2.0. "First-distances" analysis was carried out in QIIME2-longitudinal to examine beta diversity distances between successive samples from the same subject based on a distance matrix.

Spearman's rank test was used for correlation analysis, and data visualization was performed using R (v4.4.0). Co-abundance network analysis and statistical analysis of the relative abundance of genera were also carried out in R (v4.4.0), with filtering criteria set at filter_thres = 0.0005 and COR_cut = 0.4.

**References**

1. Al-Qadami G, Verma G, Van Sebille Y, Le H, Hewson I, Bateman E, Wardill H, Bowen J: **Antibiotic-Induced Gut Microbiota Depletion Accelerates the Recovery of Radiation-Induced Oral Mucositis in Rats**. *International journal of radiation oncology, biology, physics* 2022, **113**(4):845-858.

2. Segata N, Izard J, Waldron L, Gevers D, Miropolsky L, Garrett WS, Huttenhower C: **Metagenomic biomarker discovery and explanation**. *Genome biology* 2011, **12**(6):R60.

**Appendix B**

**Supplementary Table 1. Experimental Settings and Parameters of Irradiator to Construct Mouse Radiation Oral Mucositis Model**

| Radiation parameters | | | | | |  |
| --- | --- | --- | --- | --- | --- | --- |
|  | Voltage | Current | Radiation distance | Radiation rate | Filter | Field size |
| Radiation-parameters | 225kV | 17.8mA | 55.0cm | 1.5 Gy/min | Aluminium  2.00 mm | 17.8*10.7cm^2^ |

**Supplementary Table 2. VRTOG Grading Evaluation of Tongue Mucosal Tissue in Mice with Radiation-Induced Oral Mucositis**

| VRTOG classification standard | | | | |
| --- | --- | --- | --- | --- |
| VRTOG Grade | 0 | 1 | 2 | 3 |
| Appearance | No change over baseline | Erythema | Patchy mucositis | Confluent fibrinous mucositis ulceration hemorrhagenecrosis |

**Supplementary Table 3. A Modified OM Histological Scoring System**

| Microscopic assessment of OM | |
| --- | --- |
| Grade | Histopathologic manifestation |
| 0 | No radiation injury; normal mucosa |
| 1 | Focal or diffuse alteration of basal cell layer with nuclear atypia and ≤2 dyskeratotic squamous cells |
| 2 | Epithelial thinning (2-4 cell layer) and/or ≥3 dyskeratotic squamous cells in the epithelium |
| 3 | Loss of epithelium without a break in keratinization or presence of atrophied eosinophilic epithelium with/without Subepithelial vesicle or bullous formation |
| 4 | Complete loss of epithelial and keratinized cell layers; ulceration |


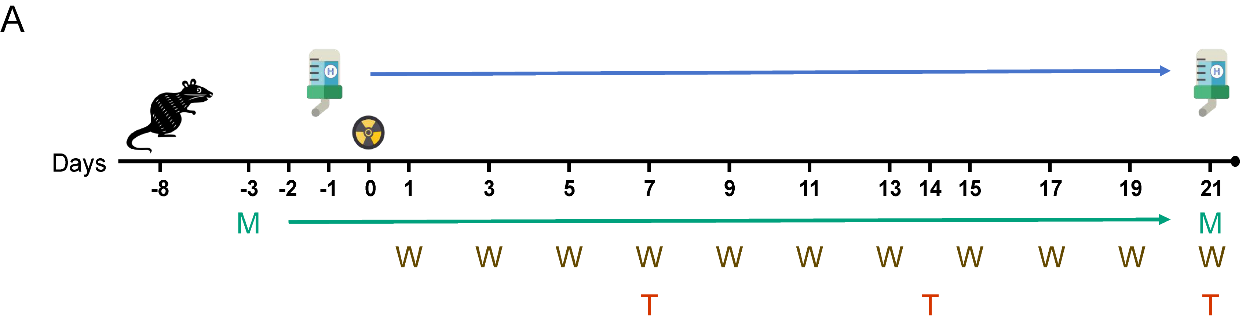


**Supplementary Figure 1. Hydrogen-Rich Water Alleviates Radiation-Induced Oral Mucositis and Its Concomitant Intestinal Inflammation in Mice.**

(A) **Experimental design scheme.** The timeline indicates key time points for various assessments. "M" represents the time point for mouth examination to evaluate oral mucositis (OM) severity. "W" represents the time point for weighing mice and collecting fecal samples for microbiota analysis. "T" represents the time point for tissue collection, including tongue and intestinal tissues, for histological evaluation.


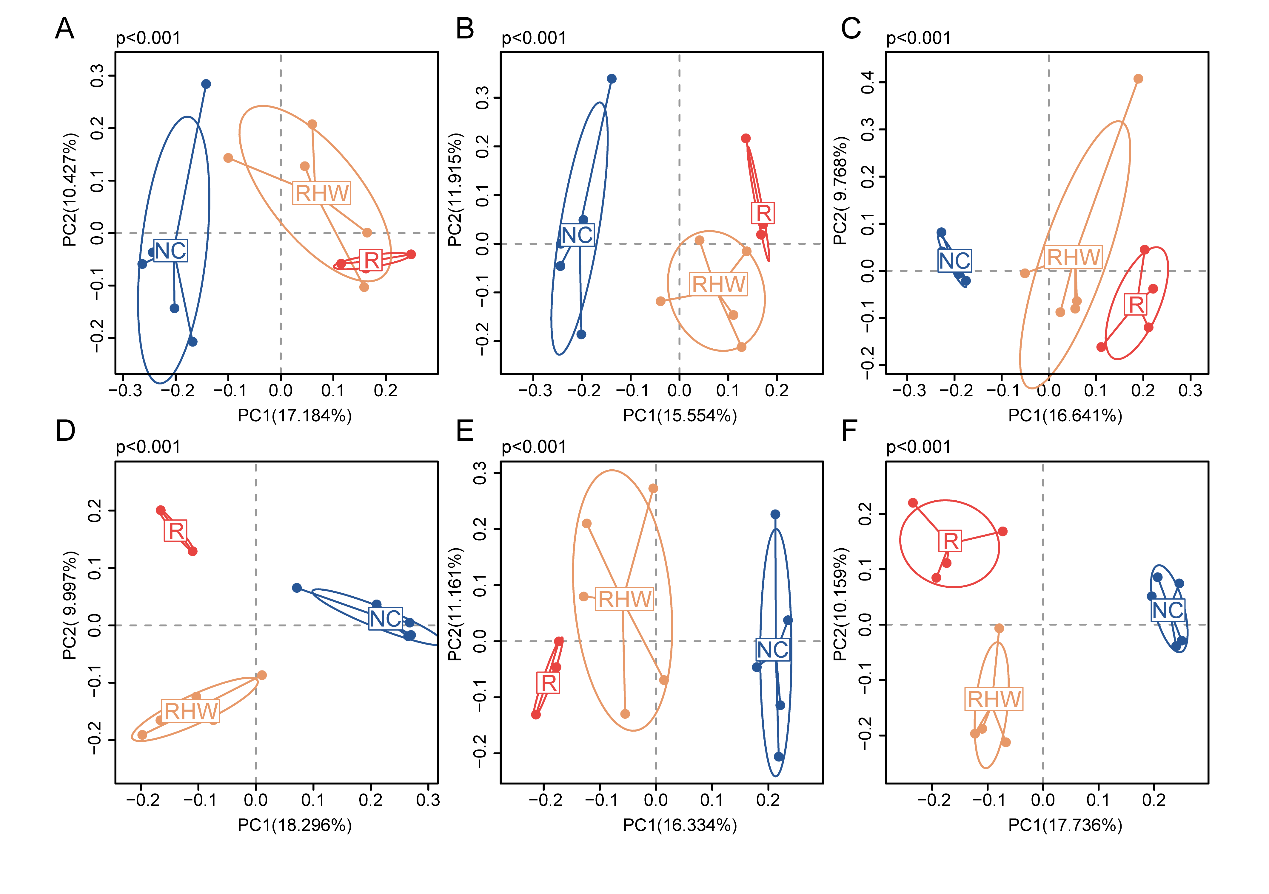


**Supplementary Figure 2. Hydrogen-Rich Water Modulates Gut Microbiota Dynamics During the Progression and Recovery of Radiation-Induced Oral Mucositis.**

(A-F) **Principal coordinate analysis (PCoA) based on unweighted UniFrac distances** was performed to compare the bacterial communities among the NC, R, and RHW groups at days 3, 5, 9, 15, and 17, respectively. Each point represents an individual sample, color-coded according to the experimental group. The diversity captured by the principal coordinates is expressed as a percentage, reflecting the differences in microbial composition between the groups at each time point.


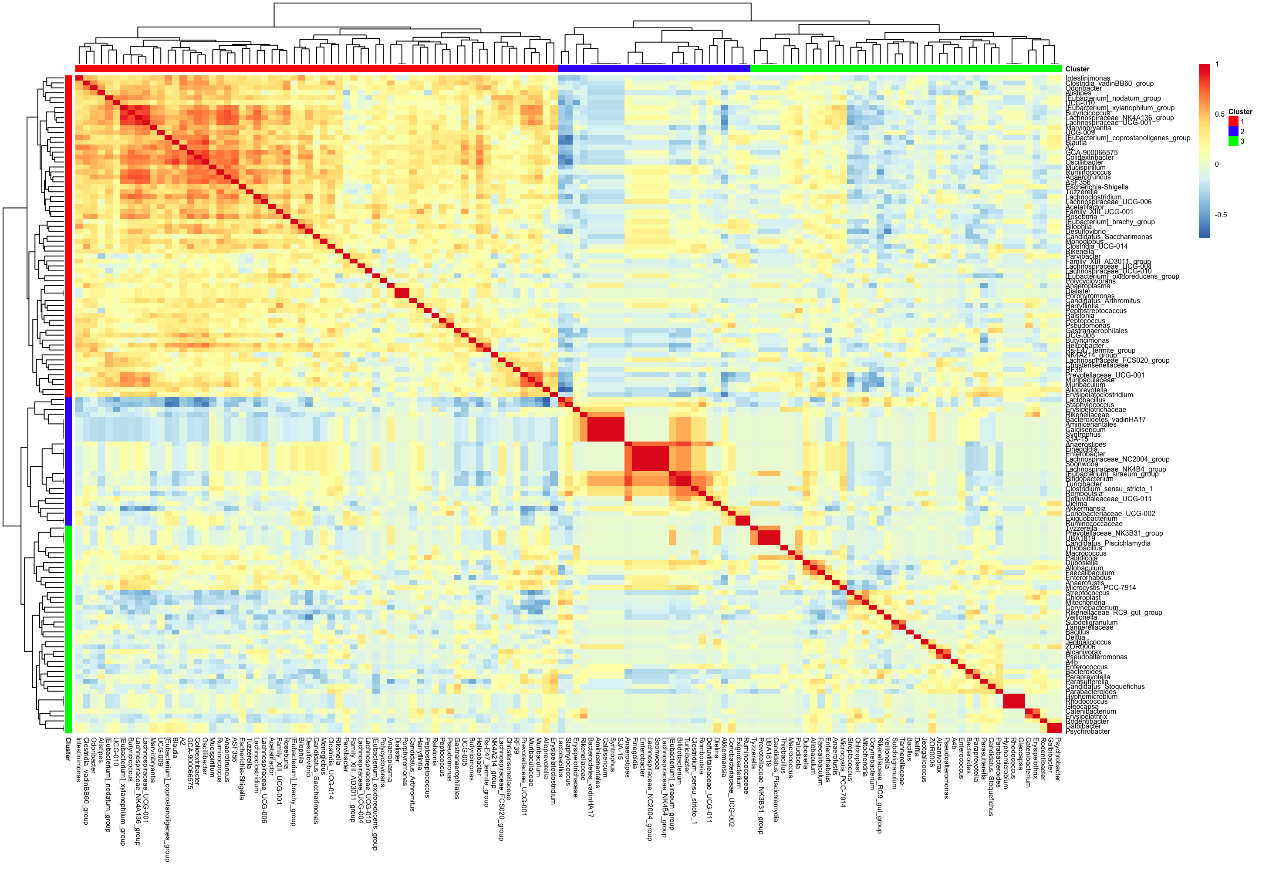


**Supplementary Figure 3. Heatmap Showing Hierarchical Clustering of Correlated Mucositis Bacterial Genera Over Time Based on Spearman Correlation Coefficients of Their Relative Abundances in the R Group.**

The heatmap displays the hierarchical clustering of mucositis-associated bacterial genera over time, with correlation values ranging from −0.55 (blue) to 1.00 (red) based on the Spearman correlation coefficients of their relative abundances. Two distinct clusters of strongly correlated genera are identified: **Cluster 1 (red)** and **Cluster 2 (blue)**. Other genera that do not fall into these clusters are marked in **green**.


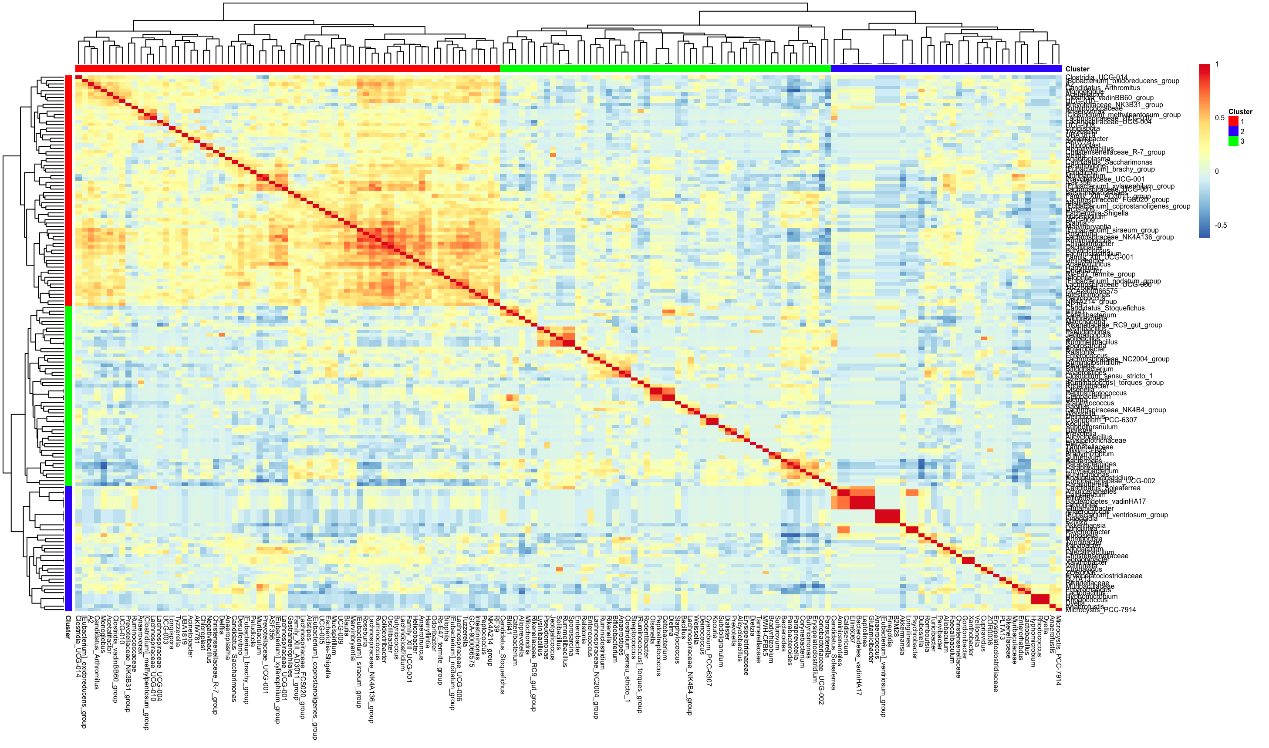


**Supplementary Figure 4. Heatmap Showing Hierarchical Clustering of Correlated Mucositis Bacterial Genera Over Time Based on Spearman Correlation Coefficients of Their Relative Abundances in the R Group.**

The heatmap illustrates the hierarchical clustering of mucositis-associated bacterial genera over time, with correlation values ranging from −0.55 (blue) to 1.00 (red) based on Spearman correlation coefficients. Two major clusters of strongly correlated genera are identified: **Cluster 1 (red)** and **Cluster 2 (blue)**. Other bacterial genera that do not fall into these clusters are marked in **green**.


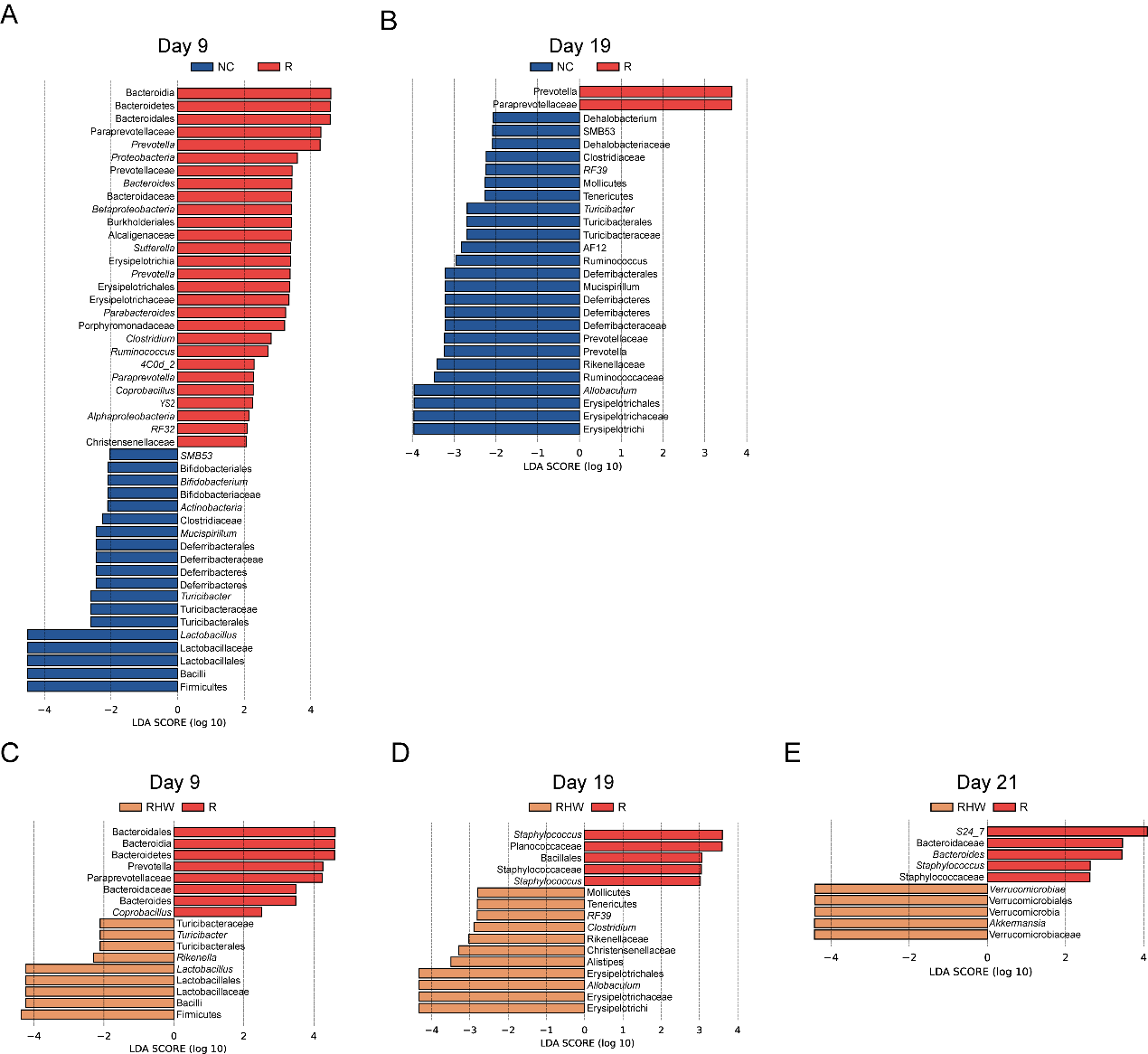


**Supplementary Figure 5. Hydrogen-Rich Water Reshapes Microbial Community Structure and Interactions in Response to Radiation-Induced Oral Mucositis.**

(A) Differences in the bacterial taxon abundance between the R and NC group in the aggravation stage by using LEfSe analysis, including Day 9. R-enriched taxa are indicated with a positive LDA score, and NC-enriched taxa have a negative score. Only taxa meeting an LDA significance threshold of >2 are shown. (B) Differences in the bacterial taxon abundance between the R and NC group in the recovery stage by using LEfSe analysis, including Day 19. R-enriched taxa are indicated with a positive LDA score, and NC-enriched taxa have a negative score. Only taxa meeting an LDA significance threshold of >2 are shown. (C) Differences in the bacterial taxon abundance between the R and RHW group in the aggravation stage by using LEfSe analysis, including Day 9. R-enriched taxa are indicated with a positive LDA score, and RHW-enriched taxa have a negative score. Only taxa meeting an LDA significance threshold of >2 are shown. (D-E) Differences in the bacterial taxon abundance between the R and RHW group in the recovery stage by using LEfSe analysis, including Day 19 (D) and Day 21 (E). R-enriched taxa are indicated with a positive LDA score, and RHW-enriched taxa have a negative score. Only taxa meeting an LDA significance threshold of >2 are shown. LEfSe, Linear discriminant analysis effect size; LDA, linear discriminant analysis.


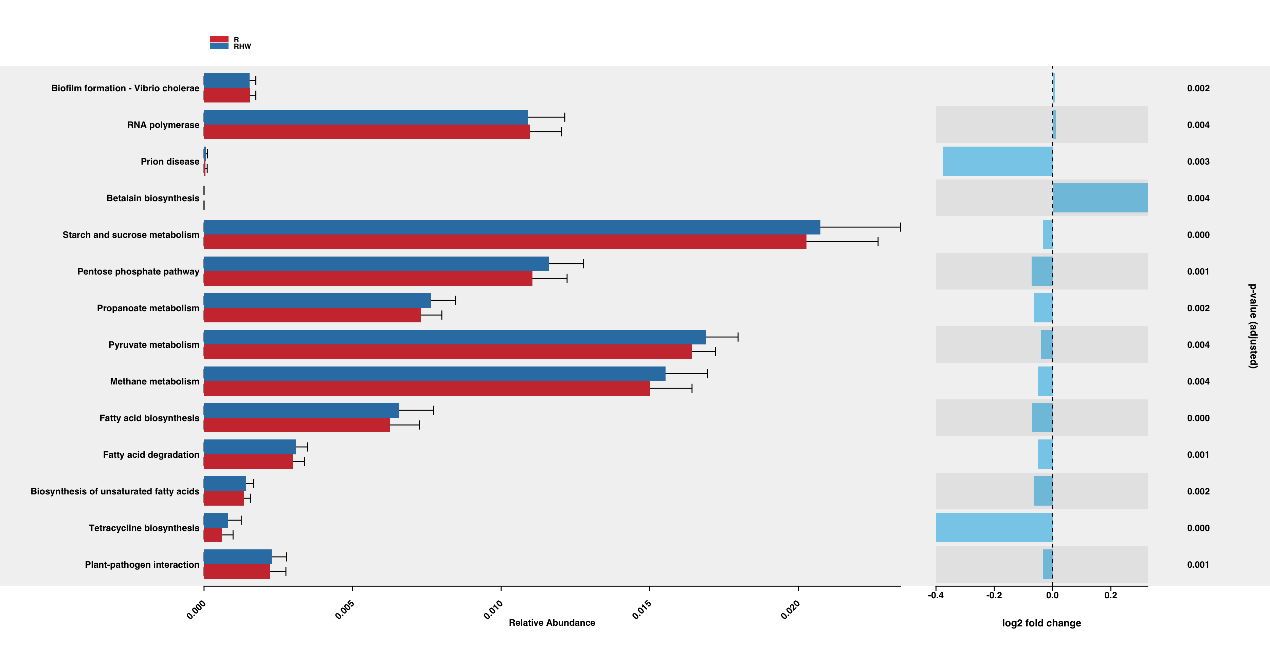


**Supplementary Figure 6. PICRUSt2-based prediction of microbial functional profiles with a focus on SCFA-related pathways.** Bar plots show the relative abundance of key KEGG pathways in the R and RHW groups. Notably, pyruvate metabolism, propanoate metabolism, and fatty acid biosynthesis were significantly enriched in the RHW group. The right panel displays log₂ fold changes and corresponding adjusted P-values.
